# Bateman's Data: Inconsistent with “Bateman's Principles”

**DOI:** 10.1002/ece3.6420

**Published:** 2020-09-14

**Authors:** Thierry Hoquet, William C. Bridges, Patricia Adair Gowaty

**Affiliations:** ^1^ Department of Philosophy University Paris Nanterre France; ^2^ Department of Mathematical Sciences Clemson University Clemson SC USA; ^3^ Department of Ecology and Evolutionary Biology University of California Los Angeles CA USA

**Keywords:** Bateman (1948), sex differences, sexual selection, variance in number of mates, variance in reproductive success

## Abstract

A.J. Bateman (1948) hypothesized that a metric of sexual selection is in sex differences of intrasexual variance in number of mates (*V*_NM_). AJB predicted that (a) males have greater variance in reproductive success (*V*_RS_) than females; (b) males have greater *V*_NM_ than females; and (c) a positive relationship between *V*_NM_ and *V*_RS_ is stronger among males. AJB used phenotypically observable mutations in offspring to identify parents and to count subjects' NM and RS. AJB's conclusions matched his predictions, later called “Bateman's Principles.” Empirical challenges to his conclusions guided analyses herein. (a) AJB's analysis pseudo‐replicated sample sizes, violating a sexual selection assumption: *That is*, individuals must be in the same population to choose and compete. (b) AJB's methods overestimated subjects with no mates while underestimating subjects with one or more. (c) A replication (Gowaty et al., 2012) showed that offspring inheriting nametags from both parents often died before expressing adult phenotypes, proving some of AJB's methods produced biased data. Science historian Thierry Hoquet located AJB's archived, handwritten laboratory notes, photocopied, and transcribed them. We tested each of the 65 unique populations for expected combinations in offspring of parental mutations: 41.5% failed Punnett's tests: Offspring carrying nametags simultaneously from both parents were missing showing estimates of parents' NM and *V*_NM_ were undercounted. 58.5% of populations met Punnett's expectations providing an unparalleled opportunity to re‐evaluate AJB's predictions. 34 unbiased populations had no sex differences in *V*_RS_; 37 had no sex differences in *V*_NM_. No sex differences in slopes of RS and NM occurred in any unbiased population. Regressions showed weak, positive, significant associations between *V*_NM_ and *V*_RS_ for females and males, contrary to AJB's prediction that the relationship would be positive in males but not in females. AJB's laboratory data are inconsistent with “Bateman's Principles.”

## Graphical abstract

Analysis of Bateman's original laboratory note data shows inconsistencies with his original hypotheses. The predictions of the hypotheses came to be known as “Bateman's Principles“.

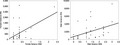

## 1 INTRODUCTION

**FIGURE 1:**
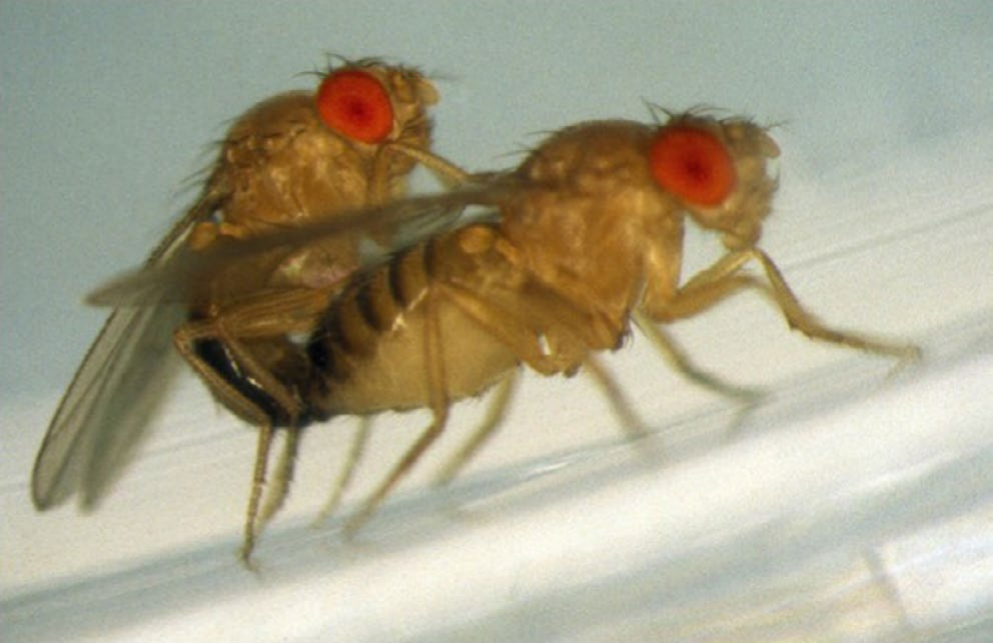
A photograph of *Drosohila melangogaster*

Bateman (A. J. Bateman, 1948) was the first laboratory experimental test of a component of sexual selection, and it is among the most cited papers in modern sexual selection. Inspired by Fisher's (1930) fundamental theorem, Bateman (1948) hypothesized that a measure of sexual selection was in the sex differences in intrasexual variances in number of mates (NM). To experimentally test this idea, he organized populations of *Drosophila melanogaster* (Figure 1) to evaluate what became known as Bateman's Principles, which are as follows: (a) Males have greater variance in reproductive success (*V*_RS_) than females; (b) variances in number of mates (*V*_NM_) for males are greater than for females; and (c) the positive relationship between *V*_NM_ and *V*_RS_ is stronger for males than females.

Here, we use the data from the handwritten laboratory notes of Angus J. Bateman (AJB), which were the basis for his published results. We use the handwritten data to re‐evaluate Bateman's predictions about sex differences in number of mates (NM), reproductive success (RS), variance in number of mates (*V*_NM_), and variance in number of offspring (*V*_RS_). Before describing our analysis methods, we review Bateman's original methods in the section *What Did Bateman Set Out to Study and What Did He Do?* Then, in “*Flies in the Ointment: Modern Challenges to Bateman ,*(1948)” we describe the literature of alternative explanations for his results, methodological errors in his published methods, and modern concerns over the implications of his conclusions, and we emphasize that the modern challenges informed our analysis methods of his laboratory notes. Thus, we did not attempt to replicate Bateman's (1948) original analysis methods because of previously identified (Gowaty, Kim, & Anderson, 2012; Snyder & Gowaty, 2007) errors in his published analysis. The last part of this section characterizes the creativity of AJB's basic experiments.

### 1.1 What did Bateman set out to study and what did he do?

AJB designed his experiment to evaluate the hypothesis that a measure of sexual selection was in the sex differences in intrasexual variance in number of mates. From this logic, he predicted that a sex difference in variance of fertility (number of offspring) was a direct measure of the sex difference in the intensity of selection (measured in terms of within‐sex differences in variances in NM and RS).

**FIGURE 2:**
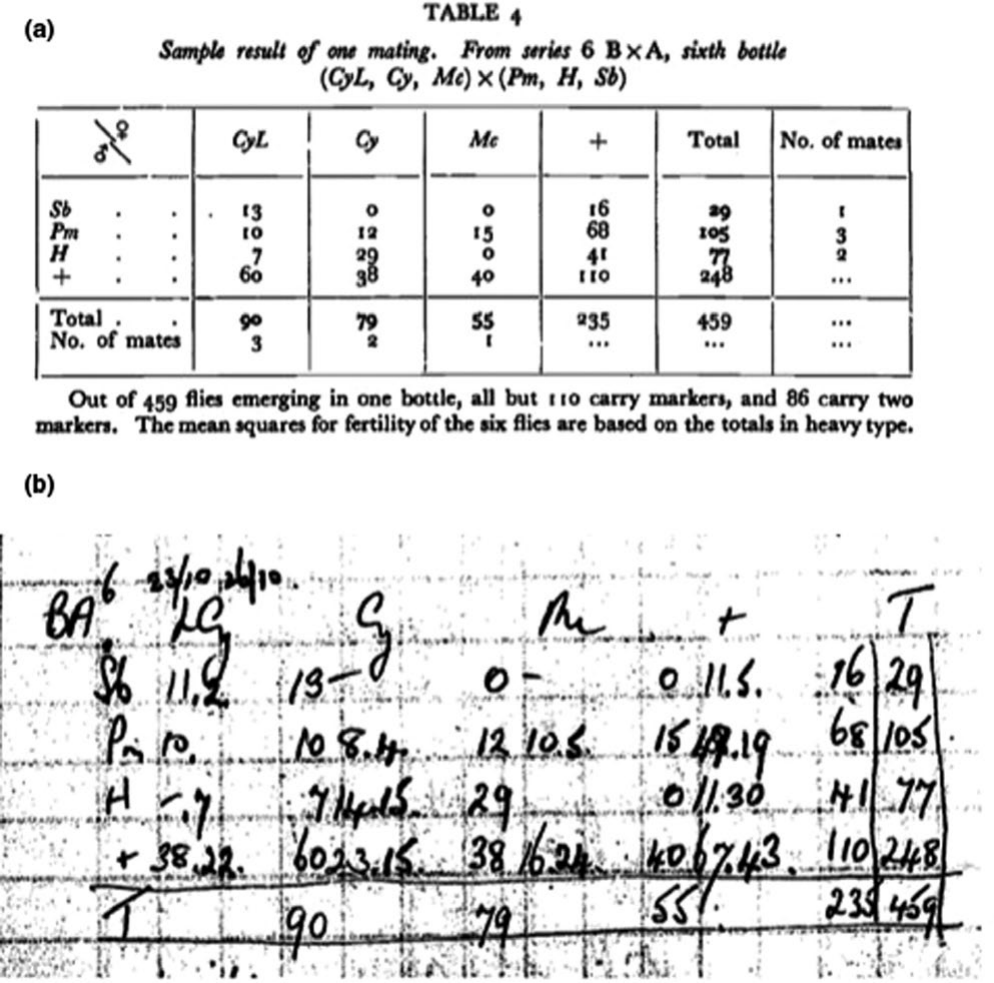
(a) The published “table 4” with its original legend from page 357 in Bateman, A. J. 1948. *Heredity* (Edinb) 2:349–368 (reprinted by permission from Macmillan Publishers Ltd. License Number: 2960430598991). Note that in both renditions of the table that only 86 (18.7%) of 459 total offspring were double‐mutant “DD” offspring, *that is*, those inheriting a nametag from each parent, significantly fewer than the frequency of 25% of total offspring required by Mendel's law (also called "Punnett's expectations.") implying that this population gave unreliable estimates of number of and variances of number of mates and reproductive success of female and male subjects. (b) A photocopy (included with permission John Innes Archives courtesy of the John Innes Foundation, used under CC‐BY 4.0 http://creativecommons.org/licenses/by/4.0/) of a data table as it appears in AJB's laboratory notes.

AJB's experiment to test the sex difference in the intensity of selection depended on complex and difficult culturing of 10 mutant fly lines to produce 10 types of heterozygote dominant subjects, each of which carried a unique identifying phenotypic marker, a “nametag,” which, when expressed in offspring, would identify the parents in each population. Table 1 is an example. It shows the relationship of each “nametag” allele among six subjects (three males and three females) illustrating that each heterozygote subject had a unique phenotypically expressed allele—a “nametag,” which when inherited in offspring would identify its parent. This method of parentage assignment provided an estimate of each subject's NM and RS. He said, *“In this way, assuming the complete viability of all the markers half the progeny of each fly would be identified”* (p. 353, Bateman, 1948), something that can readily be inferred from a Punnett square analysis (Table 2). Furthermore, AJB noted that using his method would mean that one quarter of the offspring should inherit simultaneously markers from both parents, thus providing the only estimates of the relative NM and the *V*_NM_ for male and female subjects in each population (see Figure 2a,b).

**TABLE 1:** Parental genotypes at “nametag” loci for three subjects of each sex in a sample population (redrawn from SI in Gowaty et al., 2012). The genotypes are defined by six “nametag” marker loci (Sb, Pm, H, LCy, Cy, Mc)

Adult subject	Adult genotypes (two alleles) at each marker locus					
	Sb	Pm	H	LCy	Cy	Mc
♂^1^	Sb+	++	++	++	++	++
♂^2^	++	Pm+	++	++	++	++
♂^3^	++	++	H+	++	++	++
♀^1^	++	++	++	LCy+	++	++
♀^2^	++	++	++	++	Cy+	++
♀^3^	++	++	++	++	++	Mc+

Note

Each subject was genetically and phenotypically distinct so that the dominant mutation it carried was a heritable “nametag” that, when inherited in offspring, indicated the identity of their parents. “+” indicates wildtype alleles.

**TABLE 2:** A stylized Punnett square shows combinations of nametag markers in offspring that must occur when each parent is a heterozygote dominant at a different locus

		Father's genotype	
		D_2_	+_2_
Mother's genotype	D_1_	D_1_D_2_	D_1_+_2_
	+_1_	+_1_D_2_	+_1_+_2_

Note

The subscripts are an indicator of the unique nametag loci of parents. When each parent is a heterozygote dominant at a unique nametag locus, the frequency of offspring in each cell of the Punnett square is 1/4. If the frequency of offspring in ithe cell for offspring inheriting dominant phenotypes (i.e., D_1_D_2_) from both parents is significantly less than the expected 1/4, estimates of both NM and V_NM_ would be misidenified as it was only the D_1_D_2_ offspring that provided estimates of an individiual's NM and the within population V_NM_.

AJB's chosen nametag genes produced dramatic phenotypic markers: AJB noted that 7 of 10 of the mutant lines were “homozygote lethal” (see Bateman, 1948, p. 355 for a table describing the marker genes), which was a prescient sign of the possibility that double‐mutant offspring inheriting two dramatic (different) phenotypes, one from each parent, might not always be any more viable than the homozygous lethal individuals in the originating lines expressing the identifying nametag mutations that AJB used.

Figure 2a,b shows how AJB counted the NM and the RS of each adult in each of his populations. AJB reported no observations of behavior, either in the published paper or in the laboratory notes. In each population, his inferences about sex differences in NM and RS were entirely dependent on observations in the offspring of parental nametag phenotypes. From Figure 2a,b, one can see that he noted females by their nametag phenotypes at the top of the table and males by their nametag phenotypes on the left side of the table. AJB then estimated the NM for females (bottom row) and for males (right hand column) by summing over the cells. In 14 of the populations, he used 5 males and 5 females while in later populations he reduced the number of adult males and females to 3 in each population. He pooled the 65 populations over a set of “series” (characterized by numbers of adults in each populations, the number of days individuals could mate, the distributions of nametags, combinations of ages of flies, and the pedigrees of the adult subjects) in order to calculate the mean squares for the effects of the marker phenotypes and error. He then pooled all the populations to produce a single analysis of variance. At the end of the paper, he presented two graphs, showing the “relative fertility” (RS) of females and males as a function of their numbers of mates (NM). AJB justified making two graphs of sex differences in “relative fertility” saying the populations in “series 5 and 6 differed somewhat from the rest” (Bateman, 1948, p. 361).

He concluded: “*It can now be seen that the sex difference in variance of fertility, which is itself a sign of intra‐masculine selection is due to the effect of number of mates per fly on fertility. This takes effect in two ways: ‐ (a) the higher variance, in males, of the number of mates per fly. This is a sign of intra‐masculine selection. (b) The stronger correlation, in males between number of mates and fertility. This is the cause of intra‐masculine selection”* (Bateman, 1948, p. 362).”

### 1.2 Flies in the ointment: Modern challenges to Bateman (1948)

Scholarly interest in Bateman (1948) has been a key influence on modern sex differences research, stimulating arguments claiming modern empirical consistency with AJB's conclusions despite the concerns that propelled original and critical discussions about its predictions and alternative explanations for patterns. For example, Sutherland (1985) showed that chance explains Bateman's data, a hypothesis seldom considered in recent studies of variation in NM, *V*_NM_, RS, and *V*_RS_ (however, see Hubbell & Johnson, 1987; Gowaty & Hubbell, 2005). In addition, Bateman's conclusions and the implications of his conclusions have been questioned for more than 35 years (Altmann, 1997; Gowaty & Hubbell, 2005; Hrdy, 1981, 1985, 1986, 1990; Hubbell & Johnson, 1987; Sutherland, 1985; Tang‐Martinez & Ryder, 2005). In more recent years, the quality of empirical support of Bateman's Principles has been evaluated, and discussed in relation to confirmation biases and theory tenacity (Gowaty, 2018; Tang‐Martínez, 2012, 2016). The first paper to critically evaluate Bateman's methods (Snyder & Gowaty, 2007) identified the deficit in double‐mutant offspring that was obvious in Figure 4 of Bateman (1948) (Figure 2 above). Snyder & Gowaty (2007) then speculated that AJB's methods may have seriously miscalculated *V*_NM_. To find out, Gowaty et al. (2012), Gowaty, Kim, and Anderson (2013) replicated AJB's original study using the same fly lines AJB had used. The replication (Gowaty et al., 2012, 2013) had 46 populations with 5 males and 5 females or with 3 males and 3 females similar to AJB's original set up, and it produced 8,093 offspring (Bateman's 65 populations produced 9,951 offspring). In the repetition, fathers' nametags appeared in offspring significantly more frequently than mothers' nametags, contrary to the expectation in diploid species that parental nametags should occur at similar frequencies for both sexes of parent. Additionally, Punnett's expectations were not met: Of 8,093 offspring in the 46 replicated populations, 2,343 (29%) were w♀w♂, called herein (Table 2) “++ offspring” (meaning that the offspring received only wildtype genes from each parent); 2,401 (30%) were w♀M♂ and called here “+D offspring” (meaning that from their mother–offspring received only her wildtype gene but father's nametag gene); 2,102 (26%) were M♀w♂ offspring and called here “D+ offspring”) (meaning they received their mother's nametag gene and their father's wildtype gene; and 1,247 (15%) were M♀M♂ and called here “DD offspring” (meaning that these offspring received both mother's and father's nametag genes). These frequencies were a departure from the expected 1/4 (likelihood ratio χ2 = 463.1, *df* = 3, *p* < .0001) with the biggest contribution to chi‐square coming from the double‐mutant (M♀M♂) category. In the 46 replicated populations, 44 had fewer than 20% M♀M♂, double‐mutant offspring. None of the populations had a frequency of M♀M♂s over 24.3%. The binomial probably that all 46 populations would have M♀M♂ frequencies under 25% is 1.42 Å ~ 10−14. These results proved that deficits in double‐mutant offspring were common in the mutant lines AJB used, and thus a source of miscalculation of within‐sex *V*_NM_.

AJB carefully alerted readers to a potential problem in his method of assigning NM when he said “*assuming the complete viability of all the markers, half the progeny of each fly would be identified”* (p. 353, Bateman, 1948), yet there is no evidence in his paper or in the laboratory note data that he tested the viability of the markers, either by calculation of expected distributions of types of offspring (see Table 2 above) or by monogamous control experiments (see Gowaty et al., 2012; Gowaty et al., 2013 for a description of control experiments) that had he done them, would have eliminated any possibility of intrasexual selection, but would have revealed that double‐mutant offspring were often absent, as was obvious in Bateman's original Table 4 (shown herein as Figure 2a,b). The inference from the large repetition and the monogamous control experiments (Gowaty et al., 2012, 2013) is that offspring inheriting both parental nametags often died before eclosion when parental nametags would express, thereby biasing estimates of NM and critically *V*_NM_: The repetition proved that AJB's assumption of “complete viability of all the markers” was false. Consideration of the missing offspring in the critical category of double‐mutant offspring in the repetition also proved that estimates of sex differences in *V*_NM_ overestimated the NM of individuals with zero mates while underestimating the number of individuals with one or more mates. In other words, the repetition showed that missing double‐mutant offspring would produce biases in inferences about a critical parameter of Bateman's study, *that is, V*_NM_.

### 1.3 *AJB's handwritten lab* *notes showcase simplicity and elegance in his basic experiments*

Despite criticisms of Bateman's study, it was ambitious and it remains perhaps the largest ever on sexual selection. His handwritten laboratory notes consist of 65 explicit populations with tables similar to those in Figure 2 showing the counts of inherited offspring phenotypes that identified a parent's NM and their RS. His famous text (Bateman, 1948) mentioned 64, with 63 populations included in his published analyses (TH and PAG pers. obs.) His handwritten data show explicitly that he set out to study NM, RS, and sex differences in *V*_NM_ and *V*_RS_ in each population. AJB distributed his cultured subjects, the heterozygote dominant adults, into populations so that each adult subject in a particular population expressed a unique‐in‐that‐population nametag phenotype coded by a unique dominant allele at a unique locus (Table 1). AJB recorded for each population a specific table characterizing the telltale phenotypes of all offspring expressing one or more nametags or none (Figure 2a,b). For its day, AJB's culturing method, which fashioned his ability to link *some* resulting offspring to one or both parents, was potentially a creative way to empirically test hypotheses about sex differences in RS, NM, *V*_RS,_ and *V*_NM_. However, the reliability of AJB's method of parentage assignment, just as in modern molecular genetic methods, depended on the absence of biasing factors that can be an intrinsic result of the genes offspring inherit (Gowaty et al., 2012).

### 1.4 Unbiased observations allow unbiased analysis of Bateman's hypotheses

Because Bateman archived his data and because TH located the handwritten laboratory notes, we were able to perform tests in each population in his laboratory notes of the fit of expectations of frequencies of offspring types and AJB's predictions. Our analysis herein was guided by the insights of previous evaluations and repetition of Bateman's study that we discussed in the preceding sections of this introduction. In the methods section, we further characterize the steps we took in our reanalysis of AJB's data.

## 2 METHODS

### 2.1 Finding Bateman's laboratory notes

During September 2011, TH discovered AJB's original laboratory notes about his experiment published in 1948 in *Heredity* in the archive of John Innes Institute now held in Norwich, East Anglia, UK. TH is a historian and philosopher of science. His discovery of AJB's laboratory notes was due to his interest in knowing more about the context of Bateman (1948) in order to throw new light on the way AJB devised his experimental design. TH began looking for archives and documentation first by searching for information about AJB's academic affiliation when he published his 1948 paper, which indicated that AJB was at the time of the experiment at the *John Innes Horticultural Institution, Merton*. When TH was searching for AJB's archives, the John Innes Horticultural Institution had moved from Merton (Surrey) to Norwich (Norfolk). Once in touch with the JIHI archive service, TH visited the archive and opened AJB's personal file, which contained a set of material including a bunched and unbound series of sheets of paper bearing AJB's own handwriting: These handwritten notes were the original data for AJB's 1948 publication using *Drosophila melanogaster*. After discovering the archive, TH called PAG and established our collaboration.

Our use of the original data from the John Innes Archives is courtesy of the John Innes Foundation, used under CC‐BY 4.0 (http://creativecommons.org/licenses/by/4.0/).

#### 2.1.1 Transcription of the notebooks

TH transcribed the notebooks, consisting of 103 handwritten pages, with information on 65 total populations, with original data first recoded in AJB's hand into 65 unique tables. TH transcribed each data table (see example in Figure 2a) along with AJB's marginal notes into Microsoft Word text files. We were unable to find in the original publication (Bateman, 1948) any data on two of the 65 populations in the laboratory notes (Population #s 43 and 65) in the list of populations with female and male parental NM, RS, which appears in the Results section as Table 3.

#### 2.1.2 Computerized data files

PAG recorded into JMP^©^ data files each population from the transcribed laboratory book noting the variables Bateman (1948) listed as “distinctive features” of each population. The primary data set we constructed summarizes observations AJB reported by hand in his laboratory notes as a set of 65 tables, each representing a unique population. The observations included the observable phenotypes of 20,417 adult offspring from 65 populations, representing 1,300 parental nametag combinations in the reported adult offspring from 65 populations. We devised unique names for each population using the “distinctive characteristics of each population” as described in Bateman (1948).

### 2.2 The basis of our analyses

Our analysis tactics were inspired by the multiple challenges to AJB's methods noted in Sutherland (1985), Snyder and Gowaty (2007), and Gowaty et al. (2012, 2013).

### 2.3 The steps in our analyses

Step 1: How we proved which of AJB's populations were robust to evaluation of subjects' NM, *V*_NM_, RS, and *V*_RS_.

To determine whether the data in each population reliably informed questions about the NM and RS of each subject, we used likelihood ratio chi‐square tests in each population to evaluate consistency with the expectations from a Punnett square (Table 3) of the frequencies of offspring phenotypes given possible parental genotype/phenotypes (Table 1) (see discussion in Gowaty et al. (2012, 2013)). In addition, for each population, we also tested whether the number of assigned mothers and fathers was statistically similar (Table 3) as they must be in diploid species. The outcome of these analyses are in the Results section.

AJB could infer if a subject mated with other subjects only from offspring simultaneously inheriting both of its parents' nametag genes, which we call DD offspring (Table 2). AJB estimated RS for each subject by summation of all offspring that expressed a given subject's nametag (i.e., for each female subject their DD plus their D+ offspring and for each male subject their DD plus their +D offspring). If expectations of types of offspring were met, 25% of the offspring would give a reasonable estimate with some unknown likely error of the NM for each subject. For example, if only 20%–21% of offspring were DD, between 16% and 20% of mated subjects could go unobserved, so that the number of subjects with zero mates would be overestimated and the number with more than one mate underestimated rendering estimates of the NM for each subject and the within‐sex variance in NM questionable. Thus, if offspring inheriting both parental nametag genes were ≤21% (5 populations) or if the statistical distribution indicated significantly fewer than 25% DD offspring (22 populations), we considered the data unreliable for evaluation of Bateman's predictions. Twenty‐seven of the original 65 populations failed to meet Punnett's expectations and thus cannot reliably inform inferences about AJB's predictions, so we excluded them from further analyses. Two of the 65 populations in AJB's notebooks were not reported in his published paper. One of which (population 65 in our table 3) failed Punnett's expectations and so we did not analyze it further. The other (population #43 in our table 3) was Punnett consistent, and we therefore analyzed it for its fit to Bateman's predictions.

Thirty‐eight of the 65 populations fit Punnett's expected offspring frequencies given parental genotypes/phenotypes (Table 3). Using these 38 Punnett‐consistent populations, we tested Bateman's predictions.

Step 2: How we evaluated sex differences in NM, RS, *V*_NM,_ and *V*_RS_ in each population.

We *a priori* assumed that evaluation of Bateman's Principles should occur within each population, because mate choice and within‐sex rivalries could not have occurred between individuals in different populations. Therefore, we tested the first two hypotheses using the 38 unbiased populations by determining if there were sex differences in *V*_RS_ and *V*_NM_ with two‐tailed *F* tests (Table 4 and 5). Whenever the *F* test was not computable because the variance in one sex was zero, we indicated in Table 5 that the two‐tailed *F* test was nonapplicable (NA).

We tested Bateman's third hypothesis using each of the 38 unbiased populations. We compared female and male linear slopes for the relationship between RS and NM. For each population, we estimated a model that related RS to NM, sex and the NM * sex interaction. We specifically included the NM * sex interaction term of each population to allow for different slopes for females and males. We then used ANOVA to produce *F*‐statistics to test the model terms in each population. Statistically significant within‐population NM * sex interaction terms would indicate support for Bateman's third prediction.

Step 3: How we evaluated the within‐sex relationship of *V*_NM_ to *V*_RS_ across populations.

We further tested Bateman's third hypothesis using two analyses shown in Figure 5a,b. Because variances are population level metrics (not individual observation metrics), we tested separately for females and for males the association of *V*_RS_ on *V*_NM_ by regressing RS variances from the 38 unbiased populations on NM variances from the same 38 unbiased populations. Because the *V*_RS_ and *V*_NM_ had non‐normal skewed distributions and possibly violated the assumptions of traditional regression, we also performed the regression using square‐root transformed variances and rank transformed variances. The results were similar to the regressions using the untransformed data and so we report the figure and analyses in original scale for ease of interpretation.

## 3 RESULTS

**FIGURE 3:**
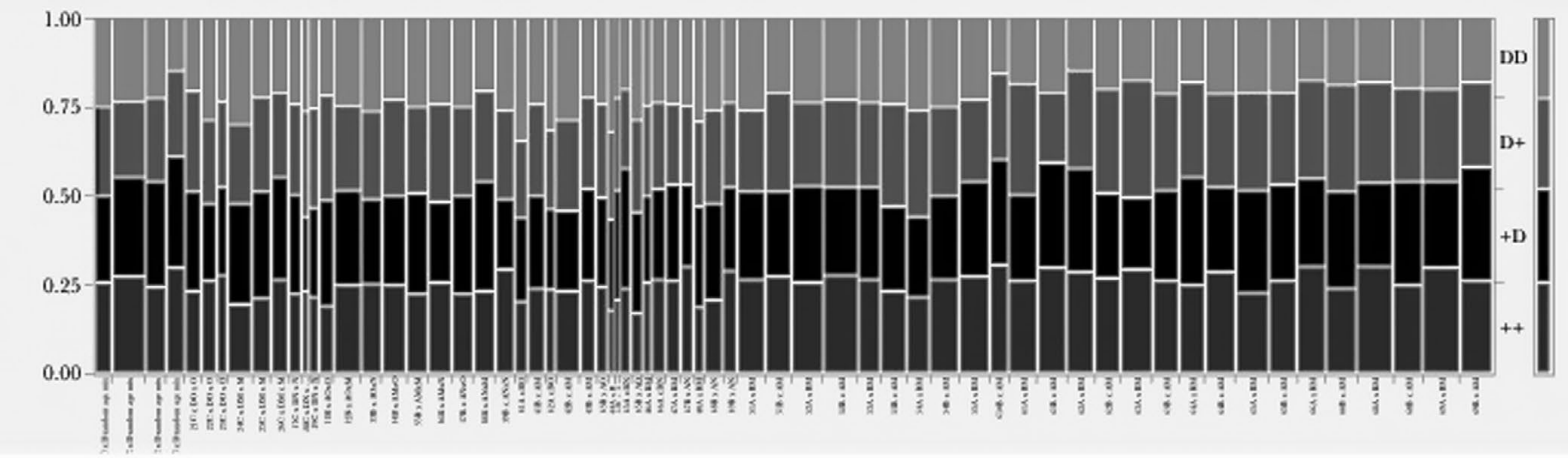
The 65 vertical lines represent one of the populations in AJB’s lab notes. The four types of offspring for each population are represented from top to bottom (DD, D+, +D, and ++ see Table 2). The light grey shading at the top of each vertical bar shows graphically that the frequencies of DD offspring were often less than the expected ¼

**FIGURE 4:**
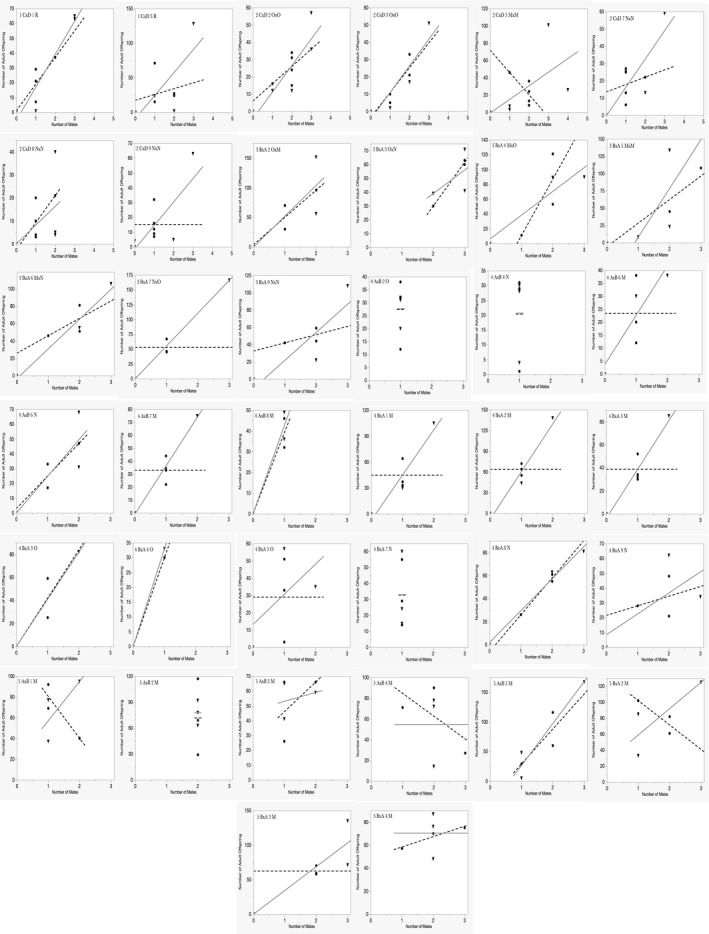
Bateman gradients for the 38 fair populations. None show significant sex differences in slopes between females and males. See statistical tests in Table 6

**FIGURE 5:**
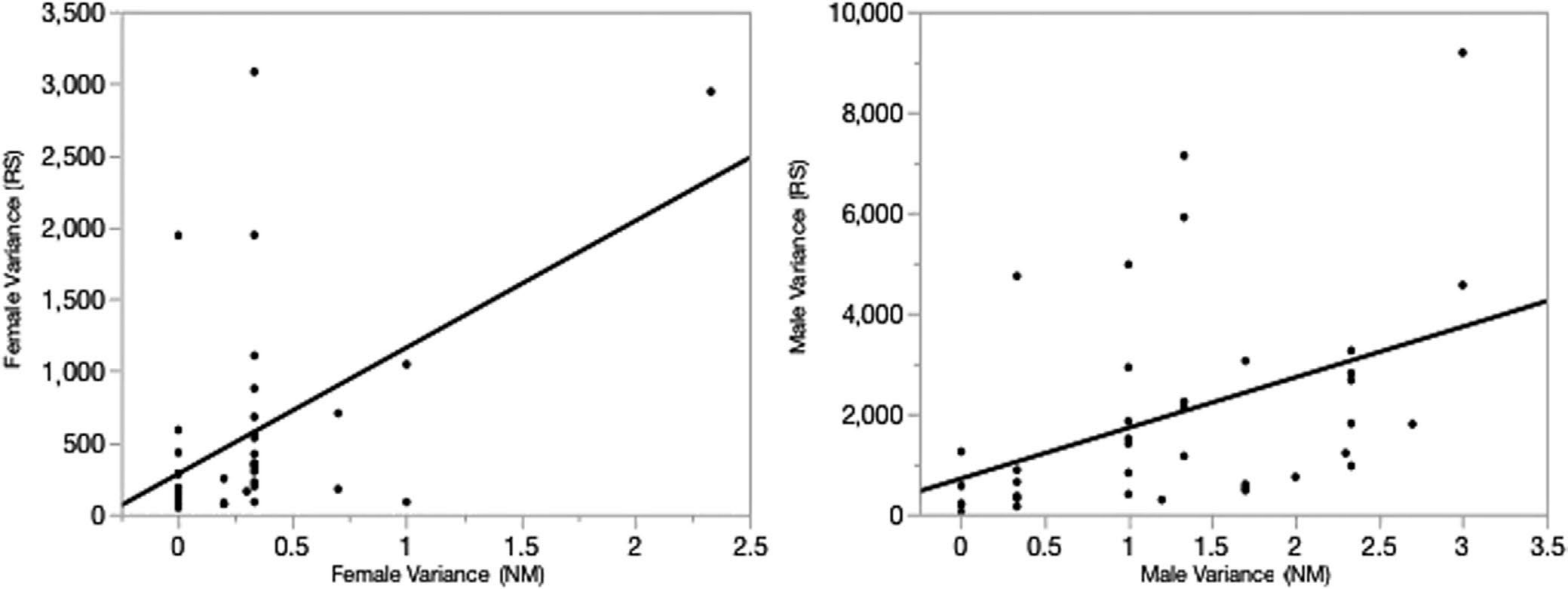
Meta‐analyses of the relationships between *V*_NM_ and *V*_RS_ for females (panel a) and for males (panel b), using the 38 unbiased populations from AJB's laboratory notes. In (a) for females, the *r* ^2^ = 0.25, *N* = 38, *df* = 37, *p* < .0013; in (b) for males, the *r* ^2^ = 0.185, *N* = 38, *df* = 37, *p* < .0070

Table 3 contains the numbers of double‐mutant (DD) offspring, D+, +D, and ++ offspring for each population and tests of Punnett's expected frequencies of types of offspring. Figure 3 summarizes the distribution of DD, D+, +D, and ++ offspring among the 20,428 offspring in the 65 laboratory note populations. Tables 4 and 5 contain tests for sex differences, respectively, in the *V*_RS_ (AJB's first prediction) and *V*_NM_ (AJB's second prediction) for each of the 38 unbiased populations. Figure 4 shows the male and female slopes relating NM and RS for each of the Punnett‐consistent populations. Table 6 contains the *F*‐ratio, and the probability of a greater *F* for comparing female and male slopes (AJB's third prediction) for each population displayed in Figure 4. The meta‐analysis of dependence of *V*_RS_ on *V*_NM_ for females is in Figure 5a and for males in Figure 5b.

### 3.1 *Tests of Punnett's expectations about DD offspring*

Thirty‐eight of the 65 populations unambiguously fit Punnett's expected offspring frequencies given parental genotypes/phenotypes (Table 3, Figure 3). AJB had included the 27 populations that did not fit Punnett's rules in his original analyses; here, we excluded the 27 biased populations from further analysis. Most of the populations in AJB's series 1–4 (which he inappropriately pooled and displayed graphically in his original paper, p. 362 fig. 1a) statistically met the expected frequency of double‐mutant offspring. However, two of the later seven populations in his “series 5” and all of the populations in his “series 6” were statistically inconsistent with one or both of Punnett's rules (equal numbers of female and male parents and ¼ DD offspring) (Table 3), yet AJB had (inappropriately) pooled these populations together to make his original fig. 1b, p. 363 (1948).

### 3.2 *Tests of the assumption that assigned mothers and fathers were statistically equal*

In diploid species, all offspring have both a mother and a father: When the frequencies of offspring that were D+ (indicating mother's identity only) and +D (indicating father's identity only) were statistically different, the estimates of RS by sex would have been inaccurate. A significant difference in the number of assigned mothers and assigned fathers could occur if the dramatic phenotypes of the nametag alleles inherited from one sex of parent were more likely to be lethal in offspring than when inherited from the other parent (Gowaty et al., 2012). Four of the 65 populations in Bateman's laboratory notes had statistically significantly different numbers of assigned fathers than mothers indicating failure to meet expectations from diploid parentage.

### 3.3 *Tests of the first and second of AJB*'*s predictions*

In four of the 38 unbiased populations, there were significant sex differences in parental *V*_RS_ (Table 4), *that is,* fewer than 11% of unbiased populations showed the predicted sex differences in *V*_RS_.

In only one of the 38 unbiased populations were V_NM_ statistically different between the sexes at the *P* < 0.05 level (Table 5), thus rejecting Bateman’s prediction about sex differences in V_NM_.

### 3.4 *Did the “Bateman gradients” show a stronger relationship of NM variances on RS variances for males than females?*

There were no significant sex differences in the slopes of the Bateman gradients (Table 6 and Figure 4) within the 38 unbiased populations. Thus, there was no evidence in any of the Punnett‐consistent populations that multiple mating by males had a greater effect on RS than it did for females providing no support for AJB's prediction.

### 3.5 *Is there a dependence of RS variances on NM variances for males but not for females?*

Because variances are population level metrics, to evaluate a dependency of *V*_RS_ on *V*_NM_ for each sex, one needs to evaluate the relationship across populations separately for females and males. Bateman expected that among males, but not among females, that the *V*_RS_ depended on the *V*_NM_. Figure 5a for females and 5b for males show the relationship of *V*_RS_ to *V*_NM_. Contrary to Bateman's predictions, there is a significant positive association between *V*_NM_ and *V*_RS_ for females and males. Note that the relationships for females and males were similar even under transformations of the variances to account for skewness and outliers. This analysis may be novel. It was possible only because AJB's work produced 38 unbiased and independent populations that could be analyzed to allow a reasonable evaluation of the within‐sex dependences of the metrics of *V*_NM_ and *V*_RS._

### 3.6 *What if most variance differences seemed higher in males?*

Given the way Bateman (1948) analyzed his data, it is tempting to consider a combined global analysis across populations of sex differences in *V*_NM_ and *V*_RS_, similar to his original analysis of variance (but see Snyder & Gowaty, 2007). Alternatives that one might find inviting to do across populations to evaluate differences in *V*_NM_ and *V*_RS_ might be a sign test or an ANOVA with population as a random effect. However, rather than a global test, we statistically tested sex differences in *V*_NM_ and *V*_RS_ for each population and reported the results in Table 4. The format of Bateman's laboratory notes—a set of stand‐alone tables describing for each population the NM and RS for each subject—had reinforced our insight that we should evaluate within‐population sex differences in *V*_NM_ and *V*_RS_ rather than pooling the data from different populations. The basic insight is that individuals must be in the same population to choose among potential mates or to compete with rivals. Thus, combining data across different populations is inconsistent with the fundamental assumptions of sexual selection.

Had we done a global analysis, we would have nevertheless needed to point out that there are some populations in which there are minimal or no sex differences in *V*_NM_ and *V*_RS_ estimates. Such populations (see Table 4) are evidence of inconsistency with Bateman's Principles, namely that the key variance differences in his study (*V*_RS_ and *V*_NM_) would—he said—always be greater in males than females. We also note that an overall analysis based on pooled populations can be misleading. An analysis combining the populations as Bateman (1948) did to test sexual selection hypotheses fails to link all the components in the inferential chain of sexual selection (as emphasized earlier). Sexual selection is a within‐population, within‐sex process in which within‐sex trait variation is associated with variances in some components of reproductive success *because* of mate choice or behavioral or physiological “competition” among same‐sex rivals that affect individual reproductive success in terms of either the numbers of offspring or the quality (viability) of offspring (Altmann, 1997). Furthermore, any “patterns” in a global analysis would potentially produce “statistical traps” because an overall conclusion about within‐population sex differential variances based on for example, difference scores across populations only works if the populations truly represent a random sample of populations, and the underlying phenomenon of interest is consistent across the populations. In other words, the assumptions necessary for a global test of Bateman's sex‐differences hypotheses using all of his populations combined were not met in his experiment.

We note that if larger samples' sizes were tested in each of the unbiased 38 populations, and the tests for sex differential variances had adequate statistical power within each of the populations; then, Bateman's data would provide more convincing evidence for his hypotheses. In addition, we also note that there are many possible alternative explanations to the patterns Bateman claimed, including potential observer bias, stochastic processes, physiological mechanisms of sexual conflict that can modify behavior of either sex, naturally occurring schedules between copulations, or the short durations of each population.

## 4 DISCUSSION

AJB's laboratory note populations that met Punnett's expectations and were thus able to reliably and fairly test Bateman's predictions provided little support for Bateman's predictions. Specifically, populations with statistically significant greater *V*_RS_ among males than females were rare (Table 4); few of the populations showed statistically significant sex differences in *V*_NM_ (Table 5); none of the unbiased populations had statistically significant sex differences in the slopes of RS on NM (Table 6; Figure 4); and the correlations between *V*_NM_ and *V*_RS_ from the 38 unbiased populations were significantly positive both for females (Figure 5a) and for males (Figure 5b).

That AJB's laboratory note data are largely inconsistent with his predictions does not imply that tests in other species or even other tests with *D. melanogaster* would be inconsistent. One simple explanation for the results we report is that the sample sizes in each population were too small to expose sex differences in fitness measures. The small sample sizes of adult subjects—3 females and 3 males or 5 females and 5 males—suggest that other studies using larger within‐population sample sizes of subject females and males may indeed show sex differences. This would be particularly true if genetic parentage identification methodologies are not associated with differentially killing some offspring, as they did in Bateman's study and in the recent repetitions that used fly lines carrying the same dramatic mutations as in Bateman's original study (Gowaty et al., 2012, 2013).

Despite the above difficulties, AJB's laboratory data do provide a very large number of populations, each of which yielded independent information about AJB's hypotheses. Because most of AJB's unbiased populations yielded data contradicting some or all of his predictions, we mention possible explanations for his findings that may be important considerations in future studies. One obvious issue emphasized previously (Gowaty et al., 2012) is associated with the pleiotropic effects of the nametag loci. Did the dramatic nametag phenotypes of Bateman's subjects have any effects on mate preferences or on within‐sex behavioral or physiological contests? Another issue is the insistence on evaluation of sex differences in NM and RS, when other measures of fitness might matter as well, such as offspring viability (Altmann, 1997), probabilities of individual subjects' survival, and probabilities of encounters with potential mates (Gowaty & Hubbell, 2009, 2012). In other words, counts of NM and RS provide only one part of the inferential chain necessary to demonstrate sexual selection: Even Bateman's 38 unbiased populations, without more information about within‐sex trait variation and without information on opposite‐sex mate preferences or within‐sex behavioral or physiological contests over access to mates, were inadequate for inferences about sexual selection in either sex. This is because, to infer sexual selection in either sex, one must (a) document or assume in the population and sex of interest the between‐individual trait variation, (b) observe the within‐population mechanisms that link the trait or traits to within‐sex competition or to opposite‐sex mate preferences, and (c) evaluate the variation in fitness that links to the trait variation among individuals competing, preferring, and mating. Put simply: AJB's logic, which focused entirely on sex differences in means and variances of NM and RS, was only potentially able to show consistency with sexual selection, and his logic ignored alternative explanations altogether.

At first glance at AJB's handwritten laboratory notes, one might infer that what originally interested AJB was the possibility of response to selection acting to increase the mating rate of individual males because of their trait variation. However, there is no evidence (PAG pers. obs.) in the 1948 publication or his laboratory notes (PAG and TH pers. obs.) that mating rate of either sex is heritable or that fertility associated with mating rate was heritable. If individual mating success is stochastic (Sutherland (1985), the variances in NM are uninteresting relative to sexual selection acting on traits affecting the mating rate of either sex. In fact, the significant results of Bateman (1948) might be attributed to stochastic demography as previously demonstrated in re‐analyses of the Bateman's original paper (Snyder & Gowaty, 2007; Sutherland, 1985). Even if one observes that mating rate is heritable, the associated fitness variances would require partitioning to account for chance effects that inevitably occur along with any deterministic effects (Hubbell & Johnson, 1987).

A further critical perspective says it is not clear why, as AJB posited, an essential “sign” of sexual selection acting on males is lower *V*_RS_ or lower *V*_NM_ in females. Because sexual selection occurs within a sex, the *V*_RS_ or *V*_NM_ between females and males need not say anything about sexual selection in the opposite sex. This is particularly easy to justify if sexual selection works through different fitness components for females and males as hypothesized 25+ years ago (Altmann, 1997). That is, female rivals may compete over mate quality rather than quantity, and female rivalries may act through different components of RS than do the rivalries of males. For example, selection may act to favor females that increase the viability of their offspring through increased access to diverse male haplotypes complementary to their own (Gowaty, 2008) something associated with enhanced offspring immunity.

## 5 CONCLUSION

The fact that AJB's original handwritten data fail to support his paradigmatic predictions of sexual selection suggests that it might be time for a re‐assessment of how to study sexual selection. Now may be the time to seriously take up the critical challenge in Altmann's (1997) hypothesis that the salient fitness measures for sexual selection differed between females and males. If Altmann is right, a simultaneous evaluation of sex differences in NM and RS would not necessarily identify sexual selection in either sex. Altmann hypothesized that the important components of fitness for females may be different from in males: *For example,* females may value the quality of mates and the quality of offspring rather than their numbers. Altmann's hypothesis has contested the conclusion of Bateman (1948) that *sex differences* in *V*_NM_ and *V*_RS_ have any implications for an understanding of sexual selection in either sex, and now may be the time to take Altmann's hypothesis seriously and to test it. There is no reason within‐sex selection need act the same way in both sexes, and whenever it does not, there is weak justification for inferring sexual selection acting on either sex by comparison of within‐population *sex differences* in fitness variances. In other words, it is valid and likely preferable to study sexual selection within each sex separately to identify the potentially different fitness components operating on individuals by sex (Gowaty, 2015, 2017). Future tests of sex‐dependent selection may profit by considering patterns of variation *within* sexes and *between* populations that differ in trait distributions, in processes of between‐sex mating choices and within‐sex behavioral or physiological rivalries, in population sizes, in other ecological and demographic constraints that individuals experience (Gowaty & Hubbell, 2009) as well as in fitness components.

**TABLE 3:** Tests in each of the lab note populations of the fit to Mendel's expectations that (a) the frequency of DD offspring is equal to ¼ (Bold values show DD significantly < 25%); (b) the number of assigned fathers and mothers is statistically similar (Bold values are statistically different from 50%)

#	Series^1^	Cross^2^	Pop ID #	Ages^4^	++	D+	+D	DD	Tot #	*p* > Chisq likelihood ratio^5^ D+ and +D were equal	% DD	Likelihood ratio^6^ X2 that DD is <0.25 *df* = 1	Tests of Mendel^7^
1	1	C × D	1	R	67	66	64	65	262	0.8608	0.2481	X2 = 0.0051, *p* > .9431	
2	1	C × D	2	R	134	106	137	113	490	**0.0464**	0.2306	X2 = 1, *p* > .3173	Failed
3	1	C × D	3	R	72	71	88	66	297	0.1517	0.2229	X2 = 1.2545, *p* > .2627	
4	1	C × D	5	R	83	68	88	41	280	0.1088	0.1464	X2 = 17.9685, ***p* > .0001**	Failed
5	2	C × D	1	O × O	57	69	68	50	244	0.9319	**0.2049**	X2 = 2.7633, *p* > .0965	<21% DD
6	2	C × D	2	O × O	60	54	51	66	231	0.7697	0.2857	X2 = 1.5254, *p* > .2168	
7	2	C × D	3	O × O	40	35	36	34	145	0.9055	0.2344	X2 = 0.1889, *p* > .6639	
8	2	C × D	4	M × M	73	81	105	111	370	0.0780	0.3000	X2 = 4.7471, ***p* > .295**	Failed
9	2	C × D	5	M × M	55	69	79	58	261	0.4109	0.2222	X2 = 1.1024, *p* > .2937	
10	2	C × D	6	M × M	73	65	78	57	273	0.6891	0.2095	X2 = 2.5727, *p* > .1087	Questionable < 21% DD
11	2	C × D	7	N × N	42	48	52	45	187	0.2153	0.2406	X2 = 0.0881, *p* > .7666	
12	2	C × D	8	N × N	24	31	22	27	104	0.2153	0.26	X2 = 0.0509, *p* > .8216	
13	2	C × D	9	N × N	31	40	36	36	143	0.9287	0.2517	X2 = 0.0023, *p* > .9615	
14	3	B × A	1	O × O	40	63	62	45	210	0.984	0.2143	X2 = 1.478, *p* > .2241	Questionable < 21% DD
15	3	B × A	2	O × M	101	96	108	100	405	0.8685	0.2469	X2 = 0.0206, *p* > .8858	
16	3	B × A	3	O × N	77	74	72	79	302	0.6666	0.2616	X2 = 0.2142, *p* > .6435	
17	3	B × A	4	M × O	92	100	94	85	371	0.3485	0.2291	X2 = 0.8803, *p* > .3481	
18	3	B × A	5	M × M	70	76	88	77	311	0.1958	0.2476	X2 = 0.0097, *p* > .9217	
19	3	B × A	6	M × N	88	95	78	83	344	0.5346	0.2413	X2 = 0.1406, *p* > .7076	
20	3	B × A	7	N × O	71	79	87	79	316	0.1745	0.2500	X2 = 0, *p* > 1	
21	3	B × A	8	N × M	72	79	97	64	312	0.1745	0.2051	X2 = 7.4573, *p* > .0587	Questionable < 21% DD
22	3	B × A	9	N × N	84	72	57	73	286	0.7389	0.2500	X2 = 0.0418, *p* > .8381	
23	4	A × B	1	O	37	39	42	62	180	0.7389	0.3444	X2 = 7.9752, ***p* > .0047**	Failed
24	4	A × B	2	O	36	34	35	48	153	0.9042	0.3137	X2 = 3.1505, *p* > .0759	
25	4	A × B	4	N	19	26	27	35	107	0.8907	0.3271	X2 = 3.157, *p* > .0738	
26	4	A × B	5	N	41	38	57	34	170	0.0505	0.2000	X2 = 2.3807, *p* > .1228	Questionable < 21% DD
27	4	A × B	6	M	36	36	34	34	140	0.8111	0.2429	X2 = 0.0383, *p* > .8448	
28	4	A × B	6	N	54	49	51	48	202	0.8415	0.2376	X2 = 0.1669, *p* > .6829	
29	4	A × B	7	M	55	48	58	51	212	0.3310	0.2406	X2 = 1.0958, *p* > .7781	
30	4	A × B	8	M	28	35	42	43	148	0.4247	0.2905	X2 = 4.0736, *p* > .2536	
31	4	B × A	1	M	64	69	70	65	268	0.9324	0.2425	X2 = 0.0801, *p* > .7771	
32	4	B × A	2	M	82	90	81	101	354	0.4912	0.2853	X2 = 2.8570, *p* > .1306	
33	4	B × A	3	M	64	63	63	54	244	1.0000	0.22	X2 = 0.1.3586, *p* > .2438	
34	4	B × A	3	O	41	44	42	40	167	0.8292	0.2395	X2 = 0.0987, *p* > .7533	
35	4	B × A	4	O	13	16	19	14	62	0.6119	0.226	X2 = 0.198, *p* > .6564	
36	4	B × A	5	O	27	41	46	46	160	0.5918	0.2875	X2 = 1.1632, *p* > .2808	
37	4	B × A	7	N	63	47	48	51	209	0.9183	0.2440	X2 = 0.0401, *p* > .8413	
38	4	B × A	8	N	57	72	74	70	273	0.8685	0.2564	X2 = 0.0595, *p* > .8073	
39	4	B × A	9	N	59	49	48	48	204	0.9191	0.236	X2 = 0.2385, *p* > .6253	
40	5	A × B	1	M	110	94	102	107	413	0.5677	0.2591	X2 = 0.1802, *p* > .6712	
41	5	A × B	2	M	118	106	124	109	457	0.235	0.239	X2 = 0.325, *p* > .5686	
42	5	A × B	3	M	88	79	86	79	332	0.5857	0.238	X2 = 0.2599, *p* > .6102	
43	5	A × B	4	M	73	101	77	87	338	0.0716	0.2574	X2 = 0.098, *p* > .7543	AJB excluded from Bateman *(1*)
44	5	A × B	5	M	123	103	119	102	447	0.2372	0.2282	X2 = 1.1574, *p* > .282	
45	5	B × A	1	M	104	106	91	79	380	0.2850	**0.2079**	X2 = 3.742, ***p* > .0531**	Failed
46	5	B × A	2	M	142	126	128	117	513	0.9001	0.2281	X2 = 1.3428, *p* > .2465	
47	5	B × A	3	M	90	112	93	94	389	0.1842	0.2416	X2 = 1.459, *p* > .7025	
48	5	B × A	4	M	112	107	98	104	421	0.5296	0.247	X2 = 0.0198, *p* > .888	
49	6	A × B	1	M	108	129	101	76	414	0.0645	0.1836	X2 = 10.4233, ***p* > .0012**	Failed
50	6	A × B	2	M	114	110	116	58	398	0.6898	0.1457	X2 = 25.9126, ***p* > .0001**	Failed
51	6	A × B	3	M	138	154	95	81	468	**0.0002**	0.1731	X2 = 16.008, ***p* > .0001**	Failed
52	6	A × B	4	M	91	99	111	65	366	0.4075	0.1776	X2 = 11.026, ***p* = .0009**	Failed
53	6	A × B	5	M	111	135	139	100	485	0.8090	0.2062	X2 = 8.8388, *p* = .0315	Failed
54	6	A × B	6	M	125	114	103	72	414	0.4551	0.1739	X2 = 15.9605, *p* = .0012	Failed
55	6	A × B	8	M	160	149	122	95	526	0.1007	0.1806	X2 = 19.9342, *p* = .0002	Failed
56	6	A × B	9	M	160	138	130	108	536	0.6250	0.2015	X2 = 10.3927, *p* = .011	Failed
57	6	B × A	1	M	129	85	130	91	435	**0.0021**	0.2092	X2 = 16.1427, *p* = .0011	Failed
58	6	B × A	2	M	100	109	89	74	372	0.1549	0.1989	X2 = 5.4424, *df* = 1, *p* > .0197	Failed
59	6	B × A	3	M	106	111	102	85	404	0.2030	0.210	X2 = 3.5106, *df* = 1, *p* > .061	Questionable < 21% DD
60	6	B × A	4	M	128	119	108	95	450	0.4652	0.211	X2 = 3.7676, *df* = 1, *p* < .0523	Failed
61	6	B × A	5	M	109	109	115	87	420	0.6285	0.2071	X2 = 4.2884, *df* = 1, *p* = .0384	Failed
62	6	B × A	6	M	110	138	125	86	459	0.4227	0.1874	X2 = 13.4040, *p* > .0038	Failed
63	6	B × A	8	M	108	113	127	85	433	0.3660	0.1963	X2 = 7.0215, ***p* = .0081**	Failed
64	6	B × A	9	M	122	111	150	84	467	**0.0156**	0.1799	X2 = 13.3921, ***p* > .0003**	Failed
65	6	B × A	10	M	87	69	84	44	284	0.2249	0.1549	X2 = 15.193, *p* = <.0001	Failed AJB excluded it from Bateman *(1*)

^1^ Series 1 and 2 had five female and five male subjects. Series 3–6 had three subjects of each sex.

^2^ Crosses indicated combinations of subject nametags A = Pm, H, and Sb; B = Cyl, Cy, and Mc; C = Hw, Pm, Sb, H, and Me; D = B, Cy, Cyl, Bl, and Mc.

^3^ Flies transferred to a new bottle every day.

^4^ Individual flies put into populations when 6 days old = O; when 3 days old = M, when 1 day old = N.

^5^ Likelihood ratio chi‐square test statistic indicates the deviation from 50% of assigned mothers and assigned fathers.

^6^ Likelihood ratio chi‐square test statistic indicates deviation of DD from ¼, *df* = 3, or as noted *df* − 1.

^7^ Tests of Mendel's assumptions “failed” if the number of assigned mothers and fathers were not equal or if the frequency of DD offspring was significantly less than 25% or both. Additionally, if the DD frequency was < 21%, we flagged the population as questionable and did not include it in the “unambiguously unbiased” populations.

**TABLE 4:** Reproductive success means and variances by sex in 38 unbiased populations

Population cross information^1^, ^2^	Mean of reproductive success for females	Mean of reproductive success for males	Variance in reproductive success for females	Variance in reproductive success for male	*F*‐statistic (*F*); degrees of freedom (*df*); and *p*‐value (*p*) for tests of equal RS variance between females and males
1C × D Random	26.20	25.80	159.20	1,216.70	*F* = 7.6426 *df* = 4,4 *p* = .0741
1C × D3Random	27.20	30.80	710.70	3,055.20	*F* = 4.3379 *df* = 4,4 *p* = .1843
2 C × D 2 O × O	24.00	23.40	73.50	523.80	*F* = 7.1265 *df* = 4,4 *p* = .0834
2 C × D 3 O × O	13.80	14.00	175.70	478.50	*F* = 2.7234 *df* = 4,4 *p* = .3553
2 C × D 5 M × M	25.40	27.40	248.80	1,795.30	*F* = 7.2158 *df* = 4,4 *p* = .0817
2 C × D 7 N × N	18.60	19.40	78.30	598.30	*F* = 7.6411 *df* = 4,4 *p* = .0742
2 C × D 8 N × N	11.60	9.80	73.30	290.20	*F* = 3.9591 *df* = 4,4 *p* = .2112
2 C × D 9 N × N	15.20	14.40	99.70	743.30	*F* = 7.4554 *df* = 4,4 *p* = .0773
3 B × A 2 O × M	65.33	69.33	1,105.3	5,909.33	*F* = 5.3462 *df* = 2,2 *p* = .3151
3 B × A 3 O × N	51.00	50.33	333.00	321.33	*F* = 1.0363 *df* = 2,2 *p* = .9822
3 B × A 4 M × O	61.67	59.67	3,081.3	2,670.33	*F* = 1.1539 *df* = 2,2 *p* = .9285
3 B × A 5 M × M	51.00	55.00	2,943.0	4,737.00	*F* = 1.6096 *df* = 2,2 *p* = .7664
3 B × A 6 M × N	59.33	53.67	358.33	2,810.33	*F* = 7.8428 *df* = 2,2 *p* = .2262
3 B × A 7 N × O	52.67	55.33	154.33	9,185.33	***F* = 59.5162** ***df* = 2,2** ***p* = .0330**
3 B × A 9 N × N	48.33	43.33	86.33	3,257.33	***F* = 37.7297** ***df* = 2,2** ***p* = .0516**
4 B × A 2 O	27.33	27.67	185.33	44.33	*F* = 4.1805 *df* = 2,2 *p* = .3861
4 A × B 4 N	20.00	20.33	301.00	226.33	*F* = 1.3439 *df* = 2,2 *p* = .8533
4 A × B 6 M	23.33	22.67	177.33	401.33	*F* = 2.2632 *df* = 2,2 *p* = .6129
4 A × B 6 N	32.33	33.00	225.33	1,159.00	*F* = 5.1435 *df* = 2,2 *p* = .3255
4 A × B 7 M	33.00	36.33	121.00	1,410.33	*F* = 11.6356 *df* = 2,2 *p* = .1580
4 A × B 8 M	26.00	28.33	556.00	644.33	*F* = 1.1589 *df* = 2,2 *p* = .9264
4 B × A 1 M	44.67	45.00	284.33	2,925.00	*F* = 10.2872 *df* = 2,2 *p* = .1772
4 B × A 2 M	63.67	60.67	72.33	4,969.33	***F* = 68.7005** ***df* = 2,2** ***p* = .0287**
4 B × A 3 M	39.00	39.00	129.00	1,812.00	*F* = 13.4733 *df* = 2,2 *p* = .1329
4 B × A 3 O	28.00	27.33	877.00	2,241.33	*F* = 2.5557 *df* = 2,2 *p* = .5625
4 B × A 4 O	10.00	11.00	300.00	363.00	*F* = 1.2100 *df* = 2,2 *p* = .9050
4 B × A 5 O	29.00	30.67	588.00	826.33	*F* = 1.4053 *df* = 2,2 *p* = .8315
4 B × A 7 N	32.67	33.00	430.33	567.00	*F* = 1.3176 *df* = 2,2 *p* = .8630
4 B × A 8 N	47.33	48.00	350.33	1,809.00	*F* = 5.1637 *df* = 2,2 *p* = .3245
4 B × A 9 N	32.33	32.00	196.33	964.00	*F* = 4.9100 *df* = 2,2 *p* = .3384
5 A × B 1 M	67.00	69.67	679.00	881.33	*F* = 1.2980 *df* = 2,2 *p* = .8703
5 A × B 2 M	71.67	77.67	1,941.3	210.33	*F* = 9.2298 *df* = 2,2 *p* = .1955
5 A × B 3 M	52.67	55.00	533.33	156.00	*F* = 3.4188 *df* = 2,2 *p* = .4526
5 A × B 4 M	62.67	54.67	1,044.3	1,249.33	*F* = 1.1963 *df* = 2,2 *p* = .9106
5 A × B 5 M	68.33	73.67	1,944.3	7,136.33	*F* = 3.6703 *df* = 2,2 *p* = .4282
5 B × A 2 M	81.00	81.67	420.33	2,128.00	*F* = 5.0626 *df* = 2,2 *p* = .3299
5 B × A 3 M	68.67	62.33	44.33	4,560.33	***F* = 102.8647** ***df* = 2,2** ***p* = .0193**
5 B × A 4 M	70.33	67.33	86.33	404.33	*F* = 17.4469 *df* = 2,2 *p* = .1094

^1^ Population indicated by combination of subject nametags and the nametag crosses. Crosses indicated combinations of subject nametags A = Pm, H, and Sb; B = Cyl, Cy, and Mc; C = Hw, Pm, Sb, H, and Me; D = B, Cy, Cyl, Bl, and Mc. Individual flies put into populations when 6 days old = O; when 3 days old = M, when 1 day old = N. Flies transferred to a new bottle every day.

^2^ Series 1 and 2 had five female and five male subjects. Series 3–6 had three subjects of each sex.

**TABLE 5:** Means and variances in number of mates by sex in 38 unbiased populations

Population cross information^1^	Number of females (*n*) that mated out of the total^2^, and the average number of mates (NM) for the females that mated	Number of males (*n*) that mated out of the total^2^, and the average number of mates (NM) for the males that mated	*F*‐statistic (*F*); degrees of freedom (*df*); and *p*‐value (*p*) for tests of equal NM mean between females and males that mated^3^	Var in NM for all females	Var in NM for all males	*F*‐statistic (*F*); degrees of freedom (*df*); and *p*‐value (*p*) for tests of equal NM variance between females and males^4^
1C × D1 Random	*n* = 5 NM = 1.4	*n* = 3 NM = 2.33	*F* = 2.5345 *df* = 1,6 *p* = .1626	0.3	2.3	*F* = 7.6667 *df* = 4,4 *p* = .0737
1C × D3 Random	*n* = 4 NM = 1.5	*n* = 3 NM = 2	*F* = 0.7143 *df* = 1,5 *p* = .4366	0.7	1.7	*F* = 2.4286 *df* = 4,4 *p* = .4112
2C × D2O × O	*n* = 5 NM = 1.8	*n* = 4 NM = 2.25	*F* = 0.8873 *df* = 1,7 *p* = .3776	0.2	1.7	*F* = 8.5000 *df* = 4,4 *p* = .0618
2C × D3O × O	*n* = 4 NM = 1.5	*n* = 3 NM = 2.0	*F* = 0.7143 *df* = 1,5 *p* = .4366	0.7	1.7	*F* = 2.4286 *df* = 4,4 *p* = .4112
2C × D5M × M	*n* = 5 NM = 1.8	*n* = 4 NM = 2.25	*F* = 0. 4,172 *df* = 1,7 *p* = .5389	0.2	2.7	***F* = 13.5000** ***df* = 4,4** ***p* = .0272**
2C × D7N × N	*n* = 5 NM = 1.2	*n* = 3 NM = 2.0	*F* = 2.5714 *df* = 1,6 *p* = .1599	0.2	1.7	***F* = 8.5000** ***df* = 4,4** ***p* = .0618**
2C × D8N × N	*n* = 5 NM = 1	*n* = 3 NM = 2	*F* = 9.000 *df* = 1,6 *p* = .0240	0.2	1.2	***F* = 6.0000** ***df* = 4,4** ***p* = .1108**
2C × D9N × N	*n* = 5 NM = 1	*n* = 2 NM = 2.5	*F* = 32.1429 *df* = 1,5 *p* = .0024	0	2	NA
3B × A2O × M	*n* = 3 NM = 1.33	*n* = 2 NM = 2.0	*F* = 2.400 *df* = 1,3 *p* = .2191	0.33	1.33	*F* = 4.000 *df* = 2,2 *p* = .4000
3B × A3O × N	*n* = 3 NM = 2.66	*n* = 3 NM = 2.66	*F* = 0.000 *df* = 1,4 *p* = 1.000	0.33	0.33	*F* = 1.000 *df* = 2,2 *p* = 1.0000
3B × A4M × O	*n* = 3 NM = 1.66	*n* = 2 NM = 2.50	*F* = 2.1429 *df* = 1,3 *p* = .2394	0.33	2.33	*F* = 7.000 *df* = 2,2 *p* = .2500
3B × A5M × M	*n* = 2 NM = 2.50	*n* = 3 NM = 1.66	*F* = 2.1429 *df* = 1,3 *p* = .2394	2.33	0.33	*F* = 7.000 *df* = 2,2 *p* = .2500
3B × A6M × N	*n* = 3 NM = 1.66	*n* = 2 NM = 2.50	*F* = 2.1429 *df* = 1,3 *p* = .2394	0.33	2.33	*F* = 7.000 *df* = 2,2 *p* = .2500
3B × A7N × O	*n* = 3 NM = 1.0	*n* = 1 NM = 3.00	NA	0	3	NA
3B × A9N × N	*n* = 3 NM = 1.66	*n* = 2 NM = 2.50	*F* = 2.1429 *df* = 1,3 *p* = .2394	0.33	2.33	*F* = 7.000 *df* = 2,2 *p* = .2500
4A × B2O	*n* = 3 NM = 1	*n* = 3 NM = 1	NA	0	0	NA
4A × B4N	*n* = 3 NM = 1	*n* = 3 NM = 1	NA	0	0	NA
4A × B6M	*n* = 3 NM = 1	*n* = 2 NM = 1.5	*F* = 1.8000 *df* = 1,3 *p* = .2722	0	1	NA
4A × B6N	*n* = 3 NM = 1.3	*n* = 2 NM = 2	*F* = 2.4000 *df* = 1,3 *p* = .2191	0.33	1.33	*F* = 4.000 *df* = 2,2 *p* = .4000
4A × B7M	*n* = 3 NM = 1	*n* = 2 NM = 1.5	*F* = 1.8000 *df* = 1,3 *p* = .2722	0	1	NA
4A × B8M	*n* = 2 NM = 1	*n* = 2 NM = 1	NA	0.33	0.33	*F* = 1.000 *df* = 2,2 *p* = 1.0000
4B × A1M	*n* = 3 NM = 1	*n* = 2 NM = 1.5	*F* = 1.8000 *df* = 1,3 *p* = .2722	0	1	NA
4B × A2M	*n* = 3 NM = 1.0	*n* = 2 NM = 1.5	*F* = 1.8000 *df* = 1,3 *p* = .2722	0	1	NA
4B × A3M	*n* = 3 NM = 1	*n* = 2 NM = 1.5	*F* = 1.8000 *df* = 1,3 *p* = .2722	0	1	NA
4B × A3O	*n* = 2 NM = 1.0	*n* = 1 NM = 2.0	NA	0.33	1.33	*F* = 4.000 *df* = 2,2 *p* = .4000
4B × A4O	*n* = 1 NM = 1.0	*n* = 1 NM = 1.0	NA	0.33	0.33	*F* = 1.000 *df* = 2,2 *p* = 1.0000
4B × A5O	*n* = 3 NM = 1	*n* = 2 NM = 1.5	*F* = 1.8000 *df* = 1,3 *p* = .2722	0	1	NA
4B × A7N	*n* = 3 NM = 1.0	*n* = 3 NM = 1.0	NA	0	0	NA
4B × A8N	*n* = 3 NM = 1.66	*n* = 2 NM = 2.5	*F* = 2.1429 *df* = 1,3 *p* = .2394	0.33	2.33	*F* = 7.0000 *df* = 2,2 *p* = .2500
4B × A9N	*n* = 3 NM = 1.667	*n* = 2 NM = 2.5	*F* = 2.1429 *df* = 1,3 *p* = .2394	0.33	2.33	*F* = 7.0000 *df* = 2,2 *p* = .2500
5A × B1M	*n* = 3 NM = 1.33	*n* = 3 NM = 1.33	*F* = 0. 000 *df* = 1,4 *p* = 1.000	0.33	0.33	*F* = 1.000 *df* = 2,2 *p* = 1.0000
5A × B2M	*n* = 3 NM = 2	*n* = 3 NM = 2	NA	0	0	NA
5A × B3M	*n* = 3 NM = 1.33	*n* = 3 NM = 1.33	*F* = 0. 000 *df* = 1,4 *p* = 1.000	0.33	0.33	*F* = 1.000 *df* = 2,2 *p* = 1.0000
5A × B4M	*n* = 3 NM = 2	*n* = 3 NM = 2	*F* = 0. 000 *df* = 1,4 *p* = 1.000	1	0	NA
5A × B5M	*n* = 2 NM = 1.67	*n* = 2 NM = 1.67	*F* = 0. 000 *df* = 1,4 *p* = 1.000	0.33	1.33	*F* = 4.000 *df* = 2,2 *p* = .4000
5B × A2M	*n* = 3 NM = 1.67	*n* = 3 NM = 1.67	*F* = 0. 000 *df* = 1,4 *p* = 1.000	0.33	1.33	*F* = 4.000 *df* = 2,2 *p* = .4000
5B × A3 M	*n* = 3 NM = 2.0	*n* = 2 NM = 3.0	NA	0	3	NA
5B × A4M	*n* = 3 NM = 2	*n* = 3 NM = 2	*F* = 0. 000 *df* = 1,4 *p* = 1.000	1	0	NA

^1^ Population indicated by combination of subject nametags and the nametag crosses. Crosses indicated combinations of subject nametags A = Pm, H, and Sb; B = Cyl, Cy, and Mc; C = Hw, Pm, Sb, H, and Me; D = B, Cy, Cyl, Bl, and Mc. Individual flies put into populations when 6 days old = O; when 3 days old = M, when 1 day old = *N*. Flies transferred to a new bottle every day.

^2^ Series 1 and 2 had five female and five male subjects. Series 3–6 had three subjects of each sex.

^3^ NA indicates the mean square error is zero and the ANOVA *F* test is not applicable.

^4^ NA indicates either the female or the male variance is zero and the two variance *F* test is not applicable.

**TABLE 6:** Tests of differences in slopes for the Bateman gradients in the 38 fair populations

Population cross information^1^, ^2^	*F*‐statistic (*F*); degrees of freedom (*df*); and *p*‐value (*p*) for tests of equal slopes for females and males^3^
1 C × D 1 Random	*F* = 0.1975 *df* = 1,6 *p* = .6723
1 C × D 3 Random	*F* = 1.02478 *df* = 1,6 *p* = .3505
2 C × D 2 O × O	*F* = 0.1742 *df* = 1,6 *p* = .69098
2 C × D 3 O × O	*F* = 0.0334 *df* = 1,6 *p* = .8609
2 C × D 5 M × M	*F* = 1.5546 *df* = 1,6 *p* = .2589
2 C × D 7 *N* × *N*	*F* = 0.7383 *df* = 1,6 *p* = .4232
2 C × D 8 N × N	*F* = 0.0516 *df* = 1,6 *p* = .8278
2 C × D 9 N × N	NA
3 B × A 2 O × M	*F* = 0.0071 *df* = 1,2 *p* = .9405
3 B × A 3 O × N	*F* = 0.3084 *df* = 1,2 *p* = .6345
3 B × A 4 M × O	*F* = 0.7892 *df* = 1,2 *p* = .4681
3 B × A 5 M × M	*F* = 0.2335 *df* = 1,2 *p* = .6766
3 B × A 6 M × N	*F* = 0.3878 *df* = 1,2 *p* = .5970
3 B × A 7 N × O	NA
3 B × A 9 N × N	*F* = 0.3567 *df* = 1,2 *p* = .6110
4 A × B 2 O	NA
4 A × B 4 N	NA
4 A × B 6 M	NA
4 A × B 6 N	*F* = 0.0099 *df* = 1,2 *p* = .9297
4 A × B 7 M	NA
4 A × B 8 M	*F* = 0.0447 *df* = 1,2 *p* = .8521
4 B × A 1 M	NA
4 B × A 2 M	NA
4 B × A 3 M	NA
4 B × A 3 O	*F* = 0.0018 *df* = 1,2 *p* = .9696
4 B × A 4 O	NA
4 B × A 5 O	NA
4 B × A 7 N	NA
4 B × A 8 N	*F* = 0.3161 *df* = 1,2 *p* = .6306
4 B × A 9 N	*F* = 0.0501 *df* = 1,2 *p* = .8436
5 A × B 1 M	*F* = 3.8592 *df* = 1,2 *p* = .1884
5 A × B 2 M	NA
5 A × B 3 M	*F* = 0.1201 *df* = 1,2 *p* = .762
5 A × B 4 M	NA
5 A × B 5 M	*F* = 0.0590 *df* = 1,2 *p* = .8306
5 B × A 2 M	*F* = 2.735 *df* = 1,2 *p* = .24
5 B × A 3 M	NA
5 B × A 4 M	NA

^1^ Population indicated by combination of subject nametags and the nametag crosses. Crosses indicated combinations of subject nametags A = Pm, H, and Sb; B = Cyl, Cy, and Mc; C = Hw, Pm, Sb, H, and Me; D = B, Cy, Cyl, Bl, and Mc. Individual flies put into populations when 6 days old = O; when 3 days old = M, when 1 day old = N. Flies transferred to a new bottle every day.

^2^ Series 1 and 2 had five female and five male subjects. Series 3–6 had three subjects of each sex.

^3^ When either the female slope, the male slope, or the residual are not defined, the *F* test is not applicable and denoted NA.

## CONFLICT OF INTEREST

None declared.

## AUTHOR CONTRIBUTION

**Thierry Hoquet:** Data curation (equal). **William C. Bridges:** Formal analysis (supporting). **Patricia Adair Gowaty:** Data curation (equal); Formal analysis (lead); Writing the manuscript (lead).

## Data Availability

The data set we constructed summarizes observations AJB reported by hand in his laboratory notes as a set of 65 tables, each representing a unique population. The observations included the observable phenotypes of 20,417 adult offspring from 65 populations, representing 1,300 parental nametag combinations in the reported adult offspring from 65 populations. We devised unique names for each population using the “distinctive characteristics of each population” as described in Bateman (1948). The original handwritten laboratory note data were in the form of 65 separate tables each describing the adult female and male nametag phenotypes in a particular population along with the inherited nametag phenotypes in offspring. Each table catalogues the number of offspring inheriting nametag phenotypes from each adult in a population. These data are available in our tables. TH and PAG have planned publishing the 65 handwritten tables in a future book.
